# Effectiveness of Nickel–Titanium Files for Retreatment of Molars Filled with Single-Cone Hydraulic Technique Using Bioceramic Sealers: An In Vitro Study

**DOI:** 10.3390/ma18061265

**Published:** 2025-03-13

**Authors:** Jane Lee, Hyeon-Cheol Kim, Timothy Kirkpatrick, David E. Jaramillo, Sang Won Kwak, Ji Wook Jeong

**Affiliations:** 1 Department of Endodontics, School of Dentistry, The University of Texas Health Science Center at Houston, Houston, TX 77054, USA; janejieun823@gmail.com (J.L.); timothy.c.kirkpatrick@uth.tmc.edu (T.K.); david.e.jaramillo@uth.tmc.edu (D.E.J.); 2 Department of Conservative Dentistry, Dental Research Institute, Pusan National University School of Dentistry, Yangsan 50612, Republic of Korea; golddent@pusan.ac.kr

**Keywords:** bioceramic sealer, file fracture, hydraulic technique, nickel–titanium file, patency, retreatment

## Abstract

Recently, the single-cone hydraulic canal filling technique using bioceramic sealers was found to hinder retreatment due to the mechanical properties of the bioceramic sealers. This study assessed the effectiveness of four nickel–titanium rotary files in removing gutta-percha and bioceramic sealer from molar root canals in vitro. Eighty-eight root canals from extracted molars were instrumented with Vortex Blue rotary files and filled with gutta-percha and bioceramic sealer using a single-cone technique. After 30 days, the filled canals were randomly divided into four groups according to the file used for re-instrumentation: ProTaper Gold (PTG), Endo ReStart (ERS), XP-3D Shaper (XPD), and HyFlex Remover (HFR). This study assessed whether root canal filling material removal and patency were achieved within a 10-min time frame, recording the time required in seconds. The rate of regaining patency and the time required to achieve patency were compared among groups using a generalized linear model. Scanning electron microscopy was used to evaluate the mechanical changes to the files after use. The patency rate of XPD and HFR was significantly higher than PTG. ERS and XPD demonstrated shorter patency times than HFR and significantly shorter patency times than PTG. SEM images revealed a varied range of reverse windings across file groups. PTG and ERS exhibited microcracks and fractured tips, while XPD and HFR did not display these mechanical alterations. The four file systems in this study displayed varying levels of effectiveness in the retreatment of root canals filled with bioceramic sealers.

## 1. Introduction

The goal of retreatment (Retx) in endodontics is to remove microorganisms, relieve symptoms, and preserve the natural tooth by addressing issues that may have caused the failure of the primary root canal treatment. Key objectives include removing sources of persistent infection such as residual bacteria, tissue, and debris [1]. Retreatment requires the removal of the previous root canal filling material and instrumenting the canal to regain access to the apical foramen [2]. While gutta-percha (GP) is soluble in various solvents and may be removed by dissolution, further negotiation of the canal may be necessary to remove any remaining obturation material in order to clean the apical portion of the canal [2,3].

Among the many types of root canal sealers, bioceramic root canal sealers (BCSs) are widely used [4]. BCSs are a good alternative to conventional endodontic sealers based on their biocompatibility, sealing ability, and involvement in the promotion of osteogenesis [5,6,7]. Nonsurgical root canal treatment using BCS and the single-cone technique demonstrated a success rate of 90.9% measuring lesion size as the prognostic factor [8]. Furthermore, BCSs have similar success rates when used with single-cone as traditional root canal filling techniques using AH Plus sealer with warm vertical condensation [9]. However, the retrievability of BCSs need improvement.

A study using micro-computed tomography to evaluate residual filling material before and after retreatment showed that no instrument successfully removed all root canal filling material, and longer retreatment times were reported for groups filled with BCS compared to AH Plus [10]. In addition, studies showed that apical patency was not regained in all samples that were filled using GP and BCS when conventional Retx techniques were used [11,12]. While many factors influence the outcome of Retx, obtaining patency is one of the most important prognostic factors [13]. Thus, clinicians need to be able to penetrate and thoroughly remove the existing root canal filling material, including sealers.

Currently, there is no ideal solvent for the retrieval of BCSs and studies reporting the efficacy of using a solvent have not been consistent [11]. One study evaluated the efficacy of using chloroform as a solvent for removal of BCS and a single GP cone [12]. Apical patency was not achieved in 20% of samples where obturation was to the WL and 70% of samples where obturation was 2 mm short of the WL [12]. Other studies showed that patency was effectively achieved when various solvents were used in teeth filled with BCS using the single-cone technique [14,15]. However, GP solvent is not an ideal solution due to its cytotoxicity and the effects of the acidic solvents on the erosion of the dentinal wall of root canals [15]. Another recent study evaluated the retrievability of various BCSs during retreatment using 6% NaOCl, 5% acetic acid, carbonated water, or no solution [16]. The results showed that Retx without a solvent resulted in the highest patency rate.

In addition to the complete removal of root canal filling, a desirable property of files used for Retx is the preservation of the root canal anatomy and resistance to cyclic and torsional fatigue while penetrating the compacted root canal filling material. Nickel–titanium (NiTi) rotary files display superior flexibility compared to stainless steel files, which is an important property for minimizing the risk of file fracture when instrumenting curved canals [17,18]. Compared to straight canals, curved canals better represent the clinical scenario when Retx is indicated. However, Retx files have been shown to have a higher incidence of perforations and file fracture when used in curved canals compared to straight canals [3,19].

Several rotary NiTi systems designed for retreatment have been introduced to improve the outcome of obturation material retrieval procedures. The Endo ReStart system comprises the Endo ReStart Opener and Endo ReStart file (ERS). Both files in this system are NiTi and have a tip designed to ease the penetration of the obturation material. However, due to the Endo ReStart system being manufactured only recently, there are no studies on this file system. There is also limited research on the HyFlex Remover (HFR), which is a single file system consisting of a heat-treated NiTi file designed to remove obturation material with a non-cutting tip. In this study, these newer files were compared to more commonly used retreatment files such as ProTaper Gold (PTG) and XP-3D Shaper (XPD). XPD features a serpentine shape with a unique alloy that allows the instrument to change shape depending on the temperature.

The primary purpose of this study was to compare the effectiveness of four NiTi file systems in retrieving single cone GP and BCS by measuring their rate of regaining patency and the time required to achieve patency. SEM imaging was used to evaluate the mechanical alteration of the rotary files used for re-instrumentation. The null hypothesis of this study was that there were no significant differences in the patency rate or time required to achieve patency among PTG, ERS, XPD, and HFR.

## 2. Materials and Methods

### 2.1. Selection of Teeth

This study was exempted by the Institutional Review Board for the Protection of Human Subjects (HSC-DB-21-0703). One hundred forty deidentified maxillary and mandibular human molar teeth were collected and stored in 0.5% sodium hypochlorite for 1 month. Cone beam computed tomography scans of the teeth were obtained using a Planmeca ProMax 3D Classic (90 kVp, 6.3 mA, 0.2 mm voxel size) (Planmeca, Helsinki, Finland). Extracted maxillary and mandibular human molars with completely formed roots were included in this study. Teeth with existing restorations, calcifications, open apices, and fractured roots were excluded. A total of 145 canals from the 140 teeth were used following the inclusion and exclusion criteria. The curvature of the mesiobuccal and mesial canals of included molars was 26.7 ± 10.5 degrees using the Schneider technique [20].

### 2.2. Root Canal Preparation and Obturation

After gaining a straight line of access, all 145 canals were sequentially negotiated using #6, #8, and #10 stainless steel hand files (FlexoFiles; Dentsply Maillefer, Tulsa, OK, USA). Patency was confirmed when a #10 FlexoFile was visible beyond the apical foramen. Apical patency was achieved in 88 canals. For these canals, the working length was established 0.5 mm short of the apical foramen. The canals were instrumented to the working length using the crown down technique with Vortex Blue (Dentsply Tulsa Dental Specialties) up to master apical size #30/0.04. All teeth were stored in Hanks’ Balanced Salt Solution (HBSS) at 37 °C in 100% humidity for 14 days.

The root canals were obturated with GP size #30/0.04 and EndoSequence BC Sealer HiFlow (Brasseler, Savannah, GA, USA) using the single-cone technique, allowing the apical 2 mm of the canal to be filled intentionally with only sealer [15]. The teeth were temporized using Cavit (3 M, St. Paul, MN, USA) and stored in HBSS at 37 °C in 100% humidity for 30 days.

### 2.3. Endodontic Retreatment and Group Designation

**Figure 1:**
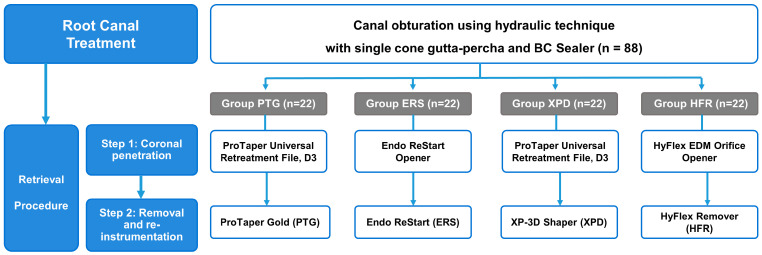
Flow chart of materials and methods.

The retrieval sequence was divided into two steps—Step 1 for coronal penetration and Step 2 for obturation material removal and re-instrumentation (Figure 1). The canals were randomly divided into 4 groups of 22 canals for Retx according to the rotary file systems used for Step 2. All Retx procedures were performed without a solvent in order to compare the mechanical properties of the file systems. An experienced endodontist performed all Retx procedures following a standardized protocol. The coronal 3 mm of GP was thermo-softened at 160 °C for 5 s using a heat plugger (Alpha A2; B&L Biotech, Fairfax, VA, USA).

Group PTG used ProTaper Universal Retreatment D3 (Dentsply Sirona, Ballaigues, Switzerland) in Step 1 and ProTaper Gold S1 (Dentsply Sirona) in Step 2. Group ERS used Endo ReStart Opener (Komet Dental, Lemgo, Germany) and Endo ReStart (Komet) for Step 1 and Step 2, respectively. Group XPD used ProTaper Universal Retreatment D3 and XP-3D Shaper for Step 1 and Step 2, respectively (Brasseler). Group HFR used HyFlex EDM Orifice Opener (Coltene, Langenau, Germany) and HyFlex Remover (Coltene) for Step 1 and Step 2, respectively. All files were used following the manufacturers’ instructions and the rotation speed (rpm; revolutions per minute) and maximum torque used are presented in Table 1. A chair-time limit of 10 min was allowed for the removal of the filling material and regaining patency. Patency was confirmed when a #10 FlexoFile was visible beyond the apical foramen. Whether patency was achieved during the time limit and, if so, the time required was recorded in seconds. The rate of patency was also calculated for each file system group.

### 2.4. Scanning Electron Microscope (SEM) Evaluation

All files used in Step 2 of Retx were placed into the SEM (SEM; JSM-7200F; JEOL, Tokyo, Japan). Images were taken under 15× and 150× magnification of different regions of the files, with 250× magnification at the file tip and for selected files, 300× magnification to evaluate areas of severe mechanical alteration.

### 2.5. Statistical Analysis

Data were analyzed using R statistical software (R Core Team 2018). A generalized linear model (GLM) at a significance level of 0.05 was used. For the patency rate and time to regain patency, a logistic regression and linear regression was performed, respectively, using Group PTG as the baseline. Statistical significance was considered at *p*-values below 0.05. Tukey’s post hoc tests were performed.

## 3. Results

**Figure 2:**
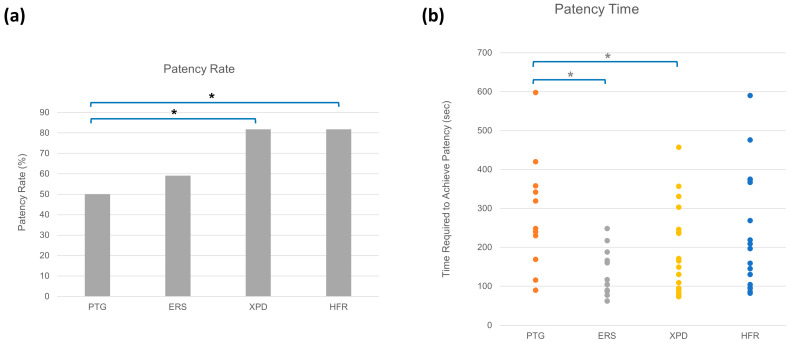
(**a**) Rate of achieving patency (%) during retreatment within the chair time limit of 10 min according to the file system used in Step 2 of the retrieval procedure. (**b**) The number of seconds needed to achieve patency for samples that successfully achieved patency within the time limit—PTG (11 canals), ERS (13 canals), XPD (18 canals), and HFR (18 canals). Asterisk (*) represents statistically significant differences among groups using Group PTG as the baseline (*p* < 0.05).

Groups XPD and HFR had the highest patency rate of 81.8%, followed by Group ERS, which had a patency rate of 59.1% (Figure 2a). Group PTG had the lowest patency rate of 50%. Using a GLM binomial distribution test, Groups XPD and HFR had significantly higher patency rates than Group PTG (*p* < 0.05). Group ERS had a higher patency rate than Group PTG, but there was no significant difference (*p* > 0.05, Figure 2a). No pair of groups was significant based on the Tukey’s post hoc analysis.

Group ERS had the lowest patency time (mean; 129.5 s) followed by Group XPD (mean; 188.3 s) and Group HFR (mean; 225.0 s). Group PTG had the longest patency time (mean; 284.5 s). Using the GLM binomial distribution test, Groups ERS and XPD had significantly lower patency times than Group PTG (*p* < 0.05, Figure 2b). Using the Tukey’s post hoc analysis, Groups PTG had a significantly lower patency time than Group ERS (*p* < 0.05).

**Figure 3:**
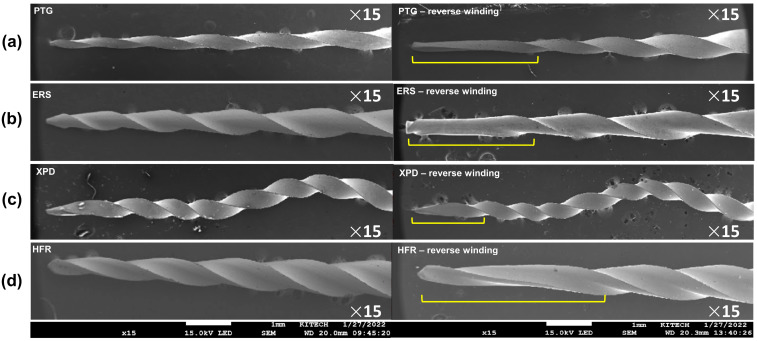
Representative scanning electron microscope images of reverse winding (yellow line) from the longitudinal aspect of the files used in Step 2 of the retrieval procedure. (**a**) PTG, ProTaper Gold; (**b**) ERS, Endo ReStart; (**c**) XPD, XP-3D Shaper; and (**d**) HFR, HyFlex Remover. HFR showed relatively longer distortion angles.

**Figure 4:**
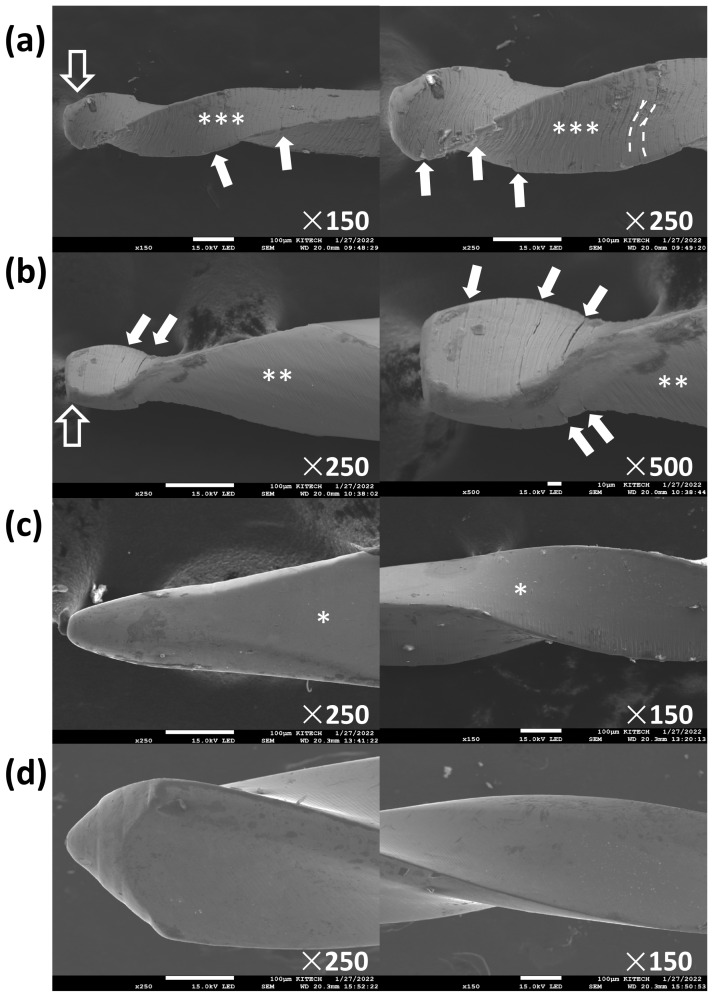
Representative scanning electron microscope images of mechanical alterations of the files after Step 2 of the retrieval procedure. (**a**) PTG, ProTaper Gold; (**b**) ERS, Endo ReStart; (**c**) XPD, XP-3D Shaper; and (**d**) HFR, HyFlex Remover. Open arrows (**a**,**b**) indicate fractured tips. Samples from PTG and ERS show microcracks (solid arrows) along the machining grooves (dotted lines). PTG shows distinct (***) machining grooves and ERS shows less distinct (**) grooves than PTG. XPD shows slight (*) machining grooves by surface treatment and HFR shows very smooth surfaces.

All file systems showed mechanical alterations of reverse windings (Figure 3). While PTG and ERS showed microcracks and broken file tips, XPD and HFR did not (Table 2, Figure 4). In terms of topographic appearance, all PTG and ERS files showed more distinct machining grooves than XPD and HFR (Figure 4). The microcracks seen in PTG and ERS were along the machining grooves (Figure 4a,b). Shaft distortion and reverse winding added some curvature to the machining grooves, which were straighter in new files (Figure 4a).

## 4. Discussion

The successful removal of root canal filling material is a critical factor in the outcome of Retx. While there are many methods to assess the successful removal of obturation material, the ability to achieve patency is considered a prognostic factor for periapical healing [13]. This is a concern for clinicians during Retx of canals filled with BCSs as studies have shown it is difficult for files to penetrate BCSs. The blockage of the apical portion of the canal with BCSs and the inability to regain patency may negatively affect the prognosis of Retx.

Previous studies showed that files designed for Retx failed to completely remove root canal filling material even when supplementary instrumentation was performed with most of the obturation material remaining in the apical portion of the canal [21,22]. Furthermore, it was reported that manual files and ProFile (Dentsply Maillefer) had better performance in removing GP compared to the Retx files used in that study [3,19]. Therefore, the present study compared the effectiveness of four file systems in retrieving GP and BCS by measuring whether patency was regained and the time to achieve patency for each canal.

Groups XPD and HFR showed significantly higher patency rates compared to Group PTG. As patency time was considered to be less relevant than patency rate when determining the effectiveness of a file, XPD and HFR were more effective in retrieving filling material compared to ERS or PTG.

In addition to its hardness upon setting, BCS displays properties of micromechanical and chemical retention to the dentinal wall, which may complicate its retrievability [23]. A previous study showed that patency was achieved in most straight, single canals that were filled with a single-cone GP and BCS, with only sealer filling the apical 1.5 mm of the canal, regardless of the solvent used [15]. However, that study included only single-rooted teeth with straight canals. Curved roots are expected to further hinder the instrumentation of canals and lead to lower patency rates [24]. Hess et al. [12] reported that when retreating mandibular molar canals that were filled with BCS using a single GP cone placed 2 mm short of the working length with the use of chloroform, patency was not achieved in 70% of samples.

The relatively higher patency rates seen in the current study may suggest that several factors should be considered when evaluating a file’s ability to negotiate a root canal that has been previously filled with BCS. Despite using molars with curved roots and no solution during retreatment in this study, patency was achieved in 50% to 81.8% of samples. This may be explained by the various mechanical factors involved in the design of NSRCT and Retx files.

Although NiTi instruments offer greater flexibility, they are susceptible to mechanical alterations due to cyclic fatigue and torsional fracture [25]. During retreatment, the files need to penetrate and remove the obturation material. Thus, the tips of files used in retreatment are subject to binding and locking into the root canal filling material [26]. This results in the tip being immobilized while the file continues to rotate. Once the torque exceeds the file’s elastic limit, torsional deformation or fracture of the instrument occurs [27]. Therefore, all the file systems in the current study showed a various range of reverse winding on the file flutes (Figure 3).

Various types of NiTi instruments that differ in cross-sectional shape, taper, and metallurgical properties have been developed to make use of the clinical advantages of NiTi instruments while addressing their risk of fracture [25,26,27]. As clinical cases involving file fractures are challenging to manage and can adversely affect the prognosis of the retreatment procedure, it is important to evaluate the mechanical and physical properties of new NiTi instruments. Thus, this study assessed the frequency of instrument distortion and fracture regardless of the effects of a solution altering the hardness of BCS as studied by Carrillo et al. [16].

The higher patency rates of XPD and HFR may be attributed to the thermomechanical treatment of these files that makes them flexible in areas of abrupt curvature where fracture is most likely [27]. The smaller initial taper (0.01) of XPD as well as the heat-treated alloy (MaxWire) may also explain its enhanced performance. In a previous study, XPD was reported to have a larger angle of rotation until fracture occurred, which means greater torsional endurance and resistance [28].

The shorter chair time necessary to achieve patency when using XPD compared to PTG and HFR may be due to the file’s ability to contact a greater surface area and increase the coronal shape of the canal, allowing the GP to be removed faster. This may be due to the adaptive core design of XPD, which allows the file to expand from an apical size #30 to #90 and increase the taper from a 0.02 to 0.08 as the working temperature changes [29]. ERS was the only file in the current study that is not heat-treated. The stiffness of ERS may be useful in the penetration of existing filling material, resulting in the lowest chair time to achieve patency. Due to its low flexibility, ERS seemed to have fewer files with unwinding after use. SEM imaging showed that PTG had more mechanical alterations compared to ERS, XPD, and HFR. Although PTG is made of heat-treated alloy, its surface texture with distinct machining grooves may result in crack initiations and propagations (Figure 4a). Furthermore, in the current study, PTG had the smallest size of all files at the apical portion. ProTaper Gold S1 has a size 18 tip and variable taper ranging from 0.02 taper at D1 to 0.11 taper at D14 [30]. As the difference in size at the apical versus coronal portion of a file increases, a higher torque may be needed to rotate the coronal portion. This can cause greater stress on the apical portion of the file and result in plastic deformation at the apical portion [30,31].

In this study, root canal filling was completed using BCS and a single cone technique, and the last 2 mm of the canals were filled with only BCS to simulate cases in which Retx is indicated. This condition of leaving only BCS in the apical portion may reduce the ability to regain patency. Thus, clinical conditions with longer GP extension close to the apex may increase the likelihood of gaining patency during Retx. Also, patency rate was solely based on the mock chair-time limit of 10 min, which is considerably shorter than the available chair-time in a clinical situation.

Patency rate was the main measure of success in this study. It is unknown whether the difference in the patency time of files is due to efficient removal of only the root canal filling material or shaping of the canal, allowing easier removal of obturation material. Future studies measuring the three-dimensional volume of the canal space after Retx are necessary to determine the efficacy of file systems to remove BCS and preserve sound dentine during Retx.

Further limitations of this study include an in vitro method, the use of one operator, and no solvent utilized in the retrieval protocol. These factors are limitations of the clinic applicability of the results. However, this study was designed to compare different file systems with a focus on their mechanical properties and eliminate factors such as variation in canal morphology, the effects of solvent facilitating the process, and the impact of operator skill and experience. Future studies should investigate how the use of solvents affects the retrievability of BCS when using the file systems used in this study.

## 5. Conclusions

Within the constraints of this study, each file system exhibited varying levels of effectiveness in restoring apical patency during the retreatment of root canals filled with bioceramic sealers. While retrieval of the bioceramic sealers applied using a single-cone hydraulic technique was feasible, the file systems assessed in this study displayed varying degrees of mechanical alterations, including reverse windings. Clinicians should take into account the mechanical characteristics of NiTi instruments when employing them for the retreatment of root canals filled with bioceramic sealers.

## Figures and Tables

**Table 1:** Sequence of file usage for gutta-percha removal and re-instrumentation for the designated group (*n* = 22).

Group	Step 1: Coronal Penetration	rpm, Ncm	Step 2: Obturation Material Removal	rpm, Ncm
PTG	ProTaper Universal Retreatment, D3 (#20/0.07)	700, 3	ProTaper Gold, S1 (#18/0.02–0.11)	700, 3
ERS	Endo ReStart Opener (#30/0.10)	700, 2.5	Endo ReStart (#25/0.05)	700, 2.5
XPD	ProTaper Universal Retreatment, D3 (#20/0.07)	700, 3	XP-3D Shaper (#30/0.02–0.08)	1200, 3
HFR	HyFlex EDM Orifice Opener (#25/0.12)	700, 2.5	HyFlex Remover (#30/0.07)	700, 2.5

**Table 2:** Mechanical alterations detected on files used for obturation material removal after use in one canal.

	Frequency of Mechanical Alterations		
Group (*n* = 22)	Reverse Winding	Microcrack	Broken Tip
PTG	10	4	9
ERS	1	1	1
XPD	6	0	0
HFR	5	0	0

## Data Availability

The original contributions presented in the study are included in the article; further inquiries can be directed to the corresponding authors.

## Abbreviations

Retx	retreatment
GP	gutta-percha
BCSs	bioceramic root canal sealers
NiTi	nickel–titanium
PTG	ProTaper Gold
ERS	Endo ReStart
XPD	XP Endo 3D Shaper
HFR	HyFlex Remover
